# Proven Cutaneous Mucormycosis in a COVID-19 Patient: A Case Report and Literature Review 

**DOI:** 10.30699/IJP.2024.2006768.3143

**Published:** 2024-02-15

**Authors:** Arezoo Salami Khaneshan, Mahsa Falahatinejad, Mahsa Abdorahimi, Mohammadreza Salehi, Farzad Aala, Alireza Abdollahi, Hana Saffar, Sadegh Khodavaisy

**Affiliations:** 1 *Department of Infectious Diseases and Tropical Medicine, Imam Khomeini Hospital complex, Tehran University of Medical Sciences, Tehran, Iran *; 2 *Department of Medical Mycology, Tarbiat Modares University, Tehran, Iran*; 3 *Department of Microbiology, Shahr‑e‑Qods Branch, Islamic Azad University, Tehran, Iran *; 4 *Research center for antibiotic stewardship and antimicrobial resistance, Tehran University of Medical Sciences, Tehran Iran*; 5 *Department of Parasitology and Mycology, Faculty of Medicine, Kurdistan University of Medical Sciences, Sanandaj, Iran*; 6 *Department of Pathology, Imam Khomeini Hospital complex, Tehran University of Medical Sciences, Tehran, Iran*; 7 *Department of Medical Parasitology and Mycology, School of Public Health, Tehran University of Medical Sciences, Tehran, Iran*

**Keywords:** COVID-19, Cuta

## Abstract

There has been a rise in COVID-19-associated mucormycosis (CAM) cases, particularly in low-income countries. We describe a case of primary cutaneous mucormycosis after recovering from COVID-19 in a kidney transplant recipient who had a known case of diabetes mellitus. The patient developed cutaneous ulcers due to Rhizopus oryzae in the right hand. She did not recall any trauma or injury at the affected site. Based on the appearance of the wound we suspected that healthcare-associated mucormycosis could be the causative agent. Due to the initial misdiagnosis as a bacterial infection, the appropriate treatment was delayed, and the lesions progressed rapidly to necrotic ulcers with jagged margins that deteriorated during hospitalization. She underwent consecutive surgical interventions and received broad-spectrum antifungal therapy. Finally, the patient deceased after 32 days of hospital stay. We reviewed the previous case reports of cutaneous mucormycosis occurring in COVID-19 patients and described patient characteristics, predisposing factors, location of ulcers, clinical presentation, management, and outcome. This report and existing published literature indicate a poor outcome for cutaneous mucormycosis in COVID-19 patients and the importance of early diagnosis, aggressive multidisciplinary management, and regular follow-up as a life-saving measure, especially in immunocompromised patients.

## Introduction

Coronavirus disease 19 (COVID-19), a viral respiratory infection with a rapid global spread, has caused the death of more than four million people globally (1). Patients with underlying comorbidities accompanying COVID-19 are more susceptible to contracting secondary bacterial or fungal infections (2). Mucormycosis has been reported as a serious infection with an unprecedented rise among patients recovering from a recent SARS-CoV-2 disease. The use of systemic glucocorticoid drugs, which play an essential role in the management of COVID-19, can also increase the risk of COVID-19-associated mucormycosis (CAM) in these patients (3). Traditional predisposing conditions for developing mucormycosis include uncontrolled and acidotic diabetes mellitus, cell or organ transplantation, extensive use of immunomodulatory drugs, malignancies, iron overload state, and other underlying diseases (4, 5). Although the most common forms of CAM are rhino-orbital-cerebral and pulmonary, *cutaneous mucormycosis* is the third most common form of this invasive fungal infection. 

*Cutaneous mucormycosis* infection is characterized by indurated plaques, tissue necrosis with surrounding erythema that can progress into an eschar, and disfigurement of limbs in severe cases (6). Primary *cutaneous mucormycosis* (PCM) involvement ensues due to the rupture of the skin barrier such as trauma, burns, surgery, or persistent maceration. It could also be rarely secondary to dissemination from internal organs to the skin (7). Herein, we describe a PCM in a kidney transplant recipient case with a history of COVID-19 and review the current literature on the subject.

## Case Report

**Fig. 1:**
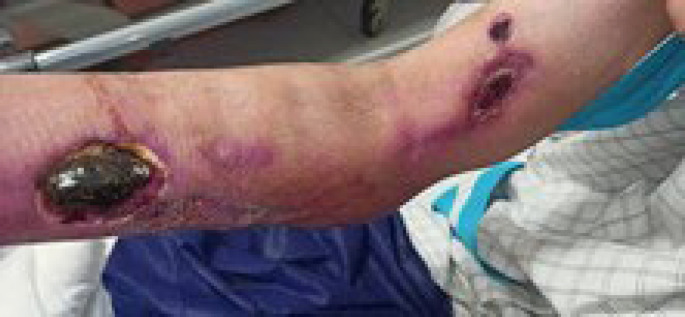
Necrotic ulcerative plaques with clear boundaries on the edge

**Fig. 2:**
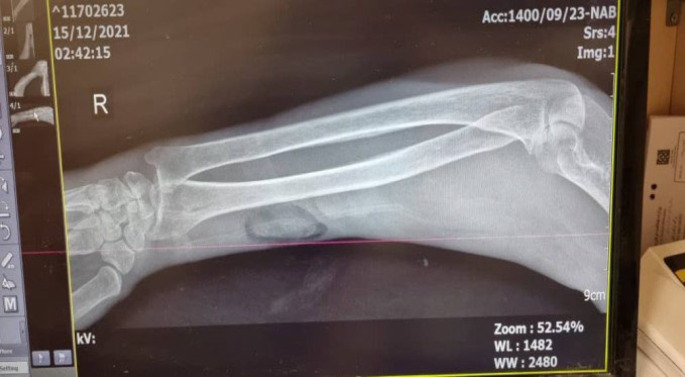
X-rays of the arm showing unremarkable bony abnormality and only showed subcutaneous skin thickening

**Fig. 3:**
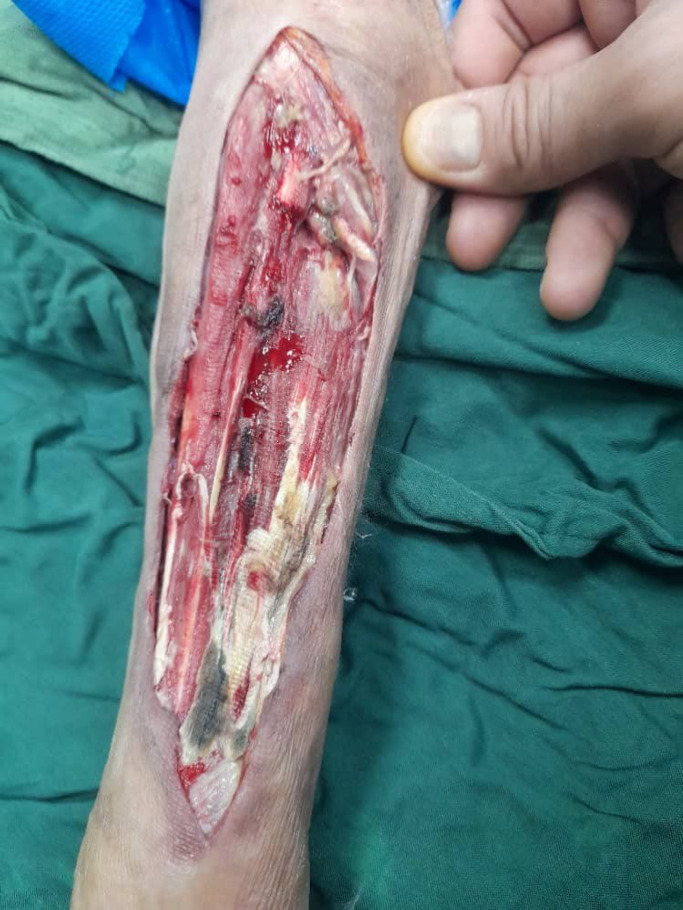
Intraoperative image of the right forearm demonstrating enlarged necrosis to the level of the muscle.

**Fig. 4:**
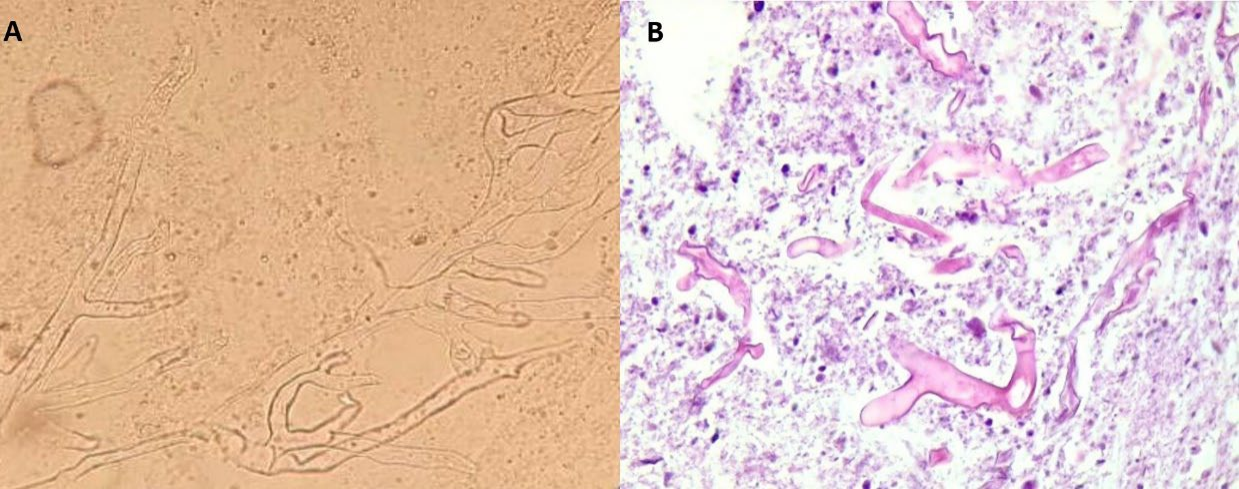
A, Direct examination of the sample with KOH 10% shows hyaline and aseptate hyphae (×400); B, Histopathologic image of the debrided lesion showing aseptate ribbon-like hyphae and giant multinucleate cells (H&E) [100X objective]

**Fig. 5:**
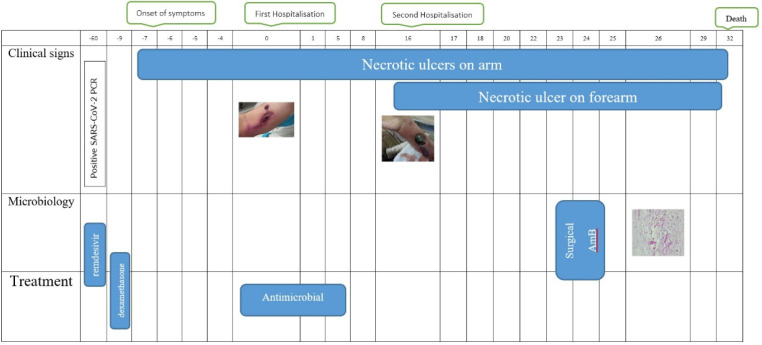
Time course of the patient with COVID–19–associated cutaneous mucormycosis

A 48-year-old female with a history of kidney transplantation 15 years before the immunosuppressive treatment with cyclosporine, Cellcept, and mycophenolate mofetil (MMF) PO 500 mg twice daily (BID), was known to have hypertension (HTN) for 20 years and diabetes mellitus for 5 years who was under treatment with insulin injection. The patient also had a deep vein thrombosis (DVT) in the lower limb, which was treated with oral rivaroxaban 15 mg daily for three months. She got a COVID-19 infection confirmed by RT-PCR test. She was admitted to a small rural hospital and treated with four doses of dexamethasone (4 mg/twice daily/IV) and remdesivir (100 mg/daily/four days). Likewise, she was hospitalized two months after recovering from COVID-19 with a skin lesion on her right arm. Based on the appearance of the lesion, a prior intravascular device adhesive site-related process was suggested. The lesion appeared as an erythematous area and gradually enlarged into a violaceous plaque measuring 5 mm in diameter. The lesion was misdiagnosed as a bacterial infection and the treatment she received included meropenem 500 mg every 12 hours, and vancomycin 1 gr every day. Although the wound was regularly washed out and dressed, it progressed to a necrotizing ulcer with tissue invasion and failed to respond to the initial treatment. She was discharged without considerable relief. Two weeks after the prior admission, she was referred to our medical emergency center with a new skin lesion on the anterior side of the right forearm. Physical examination showed a dark red irregular plaque with a purulent effusion combined with a hemorrhagic keratotic eschar in size of 7 cm in diameter (Figure 1). The patient was febrile (39 Celsius degrees) and had numbness without enlarged lymph nodes in her right hand on admission and pitting edema of the lower limbs. She denied any trauma or injury to the right arm. Clinically, the lesion was an extensive deep invasion of the tissue which seemed to follow a venous track to the forearm. Laboratory examination results were as follows: white cell count of 15.6 leukocytes/mm3 with 88.6% neutrophils and 8.4% lymphocytes and her platelet count was 300 × 103/mcl. Hemoglobin: 9.9 g/dL, C-reactive protein: 80 mg/L, ESR: 92 mm/1 h, creatinine: 1.6 μmol/L, albumin 1.9 g/L, FBS: 340 mmol/L with HbA1c: 9.1%, urea: 117 mg/dL, ferritin: 674 μg/L, total bilirubin: 0.46 mg/dL and International Normalized Ratio (INR) of 1.71. Urine analysis showed 3+ proteinuria, +3 glucose, 2-3 red blood cells (RBCs)/HP, and 3-4 pus cells/high-power field (HPF). X-ray examination revealed subcutaneous skin thickening with extension into the muscular tissue without bony abnormality (Figure 2). The pitting edema index of her lower limb values were +1 on the right and +2 on the left. Eco-Doppler displayed the existence of hyperechogenic subcutaneous tissue. The forearm ulcer was expanding with central necrosis that required surgical debridement. The initial sample obtained was sent for histopathologic and microbiologic examination (Figure 3). Based on the aggressive clinical behavior of the lesion and the clinical suspicion of the fungus, an intravenous liposomal amphotericin B (AmB) (5 mg/kg/day) injection was started empirically after the first debridement. Direct examination with potassium hydroxide (KOH) and hematoxylin and eosin (H&E) staining revealed similar features including broad non-septated hyphae with irregular wide-angle branches (Figure 4). The culture of the eschar on Sabouraud dextrose agar medium at 25˚C and 37˚C showed growth of the fluffy grayish-white colonies. Molecular analysis was achieved by 18S ITS1-5.8S-ITS2-28S region of the ribosomal DNA amplification and sequencing using the universal primers ITS1-ITS4. The consensus sequence homology was checked and compared with the GenBank database (https://www.blast.ncbi.nlm.nih.gov/ Blast. cgi). The isolated pathogen was identified as Rhizopus oryzae. According to the CLSI-M38A2 guidelines, antifungal susceptibility testing was performed. The minimum inhibitory concentrations (MICs) were as follows: amphotericin B (1 µg/mL), posaconazole (6.5 µg/mL), voriconazole (8 µg/mL), and itraconazole (2 µg/mL). The possibility of dissemination of mucormycosis was evaluated by computed tomography (CT) imaging of the sinus, chest, and abdomen, and the evidence suggestive of rhinocerebral region and pulmonary region involvement was not found. Assessment with electromyography and nerve conduction analysis revealed myopathy illness. To avoid progression of the threatening infection, cellcept was stopped and cyclosporine was tapered to 25 mg every 12 hours. The patient had several predisposing factors including diabetes mellitus, hypertension, deep vein thrombosis (DVT), prolonged steroid use, and transplanted kidney insufficiency with increased levels of creatinine. Thirty days after admission, esophagogastroduodenoscopy (EGD) reported gastrointestinal bleeding (GIB). We thought that all these factors played roles in the worsening of hemodynamic state and cutaneous mucormycosis. Unfortunately, the patient died after a 32-day hospital stay. The time course of the patient is described in Figure 5. 

**Literature review**

The English literature was reviewed for the published PCM cases using search terms “corona”, “COVID-19”, “mucormycosis”, “CAM” and “PCM”. A total of 26 cutaneous mucormycosis cases in COVID-19 patients were found. Table 1 shows the description of their clinical features, site of the lesion, predisposing risk factors, treatment, and the outcome of published PCM cases.

**Table 1:** **A **summary of the cutaneous mucormycosis cases reported in COVID-19 patients

Authors/ Reference	Country	Age /Sex	Localization	Underlying conditions	Fungal causative agents	Antifungal treatment	COVID-19 treatment	Patient outcome	Clinical presentation of Mucormycosis
Current case	Iran	48/F	Arm & forearm	Kidney transplant DM Hypertension Immunosuppressive treatment	*Rhizopusoryzae*	LAMB	Remdesivir Dexamethasone	Death	Healthcare-associated
Pragna* et al.* (1)	India	47/M	Right forearm	DM	*Mucor *spp.	AMB	Remdesivir Dexamethasone Tocilizumab	Death	Primary without trauma
Menezes* et al.* (8)	India	middle-aged man	Left axilla	Liver cirrhosis Hypertension	*Mucor* spp	LAMB + Posaconazole	Steroids therapy	Death	Primary without trauma
Tambe* et al.* (9)	India	32/M	Anterior axillary	DM	*Mucor *species	LAMB Isavuconazole voriconazole	Enoxaparin Meropenem Teicoplanin Remdesivir Ceftriaxone Prednisolone	Alive	Primary without trauma
Khatri* et al.* (10)	USA	68/M	Right axilla	Heart transplant DM Hypertension CKD Immunosuppressive therapy	*Rhizopus microspores*	LAMB Posaconazole	Plasma therapy Supportive care	Death	At intravascular device
Hammoudi* et al.* (11)	USA	60/F	Skin lesion over a patella fracture	DM	*Mucor indicus*	Voriconazole Isavuconazole posaconazole	-	Alive	Trauma
Shah* et al.* (12)	USA	94/M	The site of vaccination	Hypertension bullous Pemphigoid	*Rhizopus oryzae* complex	micafungin	-	Alive	Healthcare-associated
Annapoorani* et al.* (13)	India	Child/M	The adhesive tape site on the forearm	Chemotherapy (ALL)	*Mucorales *species	Posaconazole	Steroids therapy	Alive	Healthcare-associated
Arana* et al.* (14)	Spain	48/M	Right limb	Kidney transplant Hypothyroidism Arterial Hypertension Immunosuppressive treatment	*Lichtheimia ramosa*	LAMB+ Isavuconazole	Hydroxychloroquine Azithromycin Lopinavir Ritonavir Tocilizumab	Alive	NI
Zareshahrabadi* et al.* (15)	Iran	49/M	The right side of the head and face	Renal cancer	*Mucor *spp.	LAMB Itraconazole	Remdesavir Dexamethasone Interleukin antagonists	Alive	Trauma
Arora* et al.* (16)	India	75/M	Eschar on abdomen	Chronic obstructive pulmonary disease	*Mucorales *species	LAMB	Steroids therapy	Death	Burn
	India	38/M	Left shoulder	Diffuse large B cell lymphoma Immunosuppressive treatment	*Mucorales *species	LAMB	Steroids therapy	Alive	Trauma
	India	68/M	Dorsum of the right foot	DM Hypertension	*Mucorales *species	LAMB	Steroids therapy	Alive	Trauma
	India	42/F	Lower lip	Without prior comorbidities	*Mucorales *species	LAMB	Steroids therapy	Alive	Healthcare-associated
	India	62/M	Left nasolabial fold	DM Heart disease	*Mucorales *species	LAMB	Steroids therapy	NI	SCM
Patel* et al.* (17)	India	61/F	The right side of the face	Without prior comorbidities	*Rhizopus *species	LAMB	Remdesavir antiviral	Alive	SCM
Farid* et al.* (18)	Iraq	53/M	Skin lesion below the right eye	DKA Hypertension Acute kidney injury	*Mucorales *species	AMB	Favipiravir Anticoagulants Steroids therapy	Death	SCM
Roushdy* et al.* (19)	Egypt	59/F	Forehead and right cheek	DM	*Mucorales *species	-	Corticosteroids	Death	SCM
Saad* et al.* (20)	Egypt	44/M	Left cheek	Without prior comorbidities	*Mucorales *species	AMB	NI	Death	SCM
Belgaumkar* et al. *(7)	India	58/M	Ulcer over the left nostril right side of the Upper lip	DM	*Mucorales *species	LAMB	Remdesavir Methylprednisolone	Alive	SCM
Hamed* et al.* (21)	Palestinian	34/F	Left cheek	DKA	*Mucorales species*	AMB	Steroids therapy	Death	SCM
Nehara* et al.* (22)	India	62/F	Right upper and lower lid	DM Hypertension	*Rhizopus* species	LAMB	Steroids therapy	Alive	SCM
	India	68/F	Facial	DM	*Rhizopus arrhizus*	LAMB	Steroids therapy	Death	SCM
Chauhan* et al.* (23)	India	44/M	Left cheek	DM	*Rhizopus arrhizus*	LAMB	Steroids therapy	Death	SCM
Mahler* et al.* (24)	Romania	51/M	Facial	DM	*Mucorales *species	-	Steroids therapy Dexamethasone Dalteparin	Death	SCM
Riad* et al.* (25)	Egypt	66/M	Facial	DM Hypertension	*Mucorales *species	LAMB	Azithromycin Dexamethasone Salbutamol Sulphate, Paracetamol Acetaminophen	Alive	SCM
	Egypt	52/M	Right external nasal wall	DM Cardiovascular disease	*Mucorales *species	LAMB	Azithromycin Dexamethasone Salbutamol Sulphate, Paracetamol Acetaminophen	Alive	SCM

**Abbreviations:** ALL: Acute lymphoblastic leukemia; CKD: chronic kidney disease; DM: diabetes mellitus; DKA: diabetic ketoacidosis; F: female; LAMB: liposomal Amphotericin B; M: male; SCM: Secondary cutaneous mucormycosis

## Discussion

Mucormycosis is a serious infection among patients recovered from a recent SARS-CoV-2 infection, especially in low-income countries (25). Disruption of the skin barrier, burns, trauma, intravascular devices, catheters, or contaminated bandages is the most important predisposing factor for PCM (10). Secondary cutaneous mucormycosis (SCM) is developed from the local invasion or hematogenous dissemination to the skin from an internal source (20). Pooled data from this study indicated most patients had a history of corticosteroid usage either as chronic immunosuppression or for COVID-19 treatment. Nevertheless, corticosteroids have a multifaceted effect among COVID-19 patients. They can reduce allergic reactions, however, in combination with SARS-CoV-2 infection they can dysregulate the immune system and widely inhibit immune responses (26). Also, the hypoxia results in acidic pH and the generation of reactive oxygen species (ROS), which could lead to cellular deterioration and contribute to conditions favorable for mucormycosis proliferation (2). In our reviewed cases, the majority of the confirmed cutaneous mucormycosis in COVID-19 patients have been reported from India (14/27, 52%), followed by Egypt (4/27, 15%), USA (3/27, 11%), Iran (2/27, 7.4%), one case (1/27, 3.7%) each from Spain, Palestine, Romania and Iraq. The average age was almost 52 ± 16.4 years, and similar to the results of other studies, men (70%) constituted a more significant proportion (25). Indeed, our literature review indicated that the median time between the onset of COVID-19 and the appearance of cutaneous lesions spanned a wide range from 10 days to 4 months, and that duration likely varies by the individuals’ conditions. It is proposed that it may depend on several factors including preexisting diseases, prolonged ICU admission, the severity of COVID-19, species of the isolated fungi, and the extent, and depth and lesion sites. Also, indiscriminate use of steroids and systemic antibiotics could be other factors that were mainly linked to this issue (15,27). 

Hospitals were overwhelmed by patients during the COVID-19 pandemic and access to healthcare services could be extremely curtailed, especially in low-income countries where access to immediate identification methods was limited. This condition contributed to a delay in the cutaneous mucormycosis diagnosis. In addition, the decisive diagnosis of mucormycosis is challenging and the non-specific signs and symptoms of similar diseases may add to the intricacy of this infection diagnosis (3,14,28). Initially, in our case, the lesions were misdiagnosed as bacterial infections and did not respond to the antibiotic therapy. However, the infection flared due to the administration of prolonged steroid use, delay in diagnosis, and improper treatment.

Our review results showed that all the diagnosed patients had one or more underlying diseases, except three immunocompetent cases, and COVID-19 was the only underlying comorbidity (16,17,20). Diabetes mellitus (66.6%) was found to be one of the predominant predisposing factors, in which two cases had diabetic ketoacidosis (18,21) and three of them developed diabetes mellitus for the first time after contracting COVID-19 (17,21,24). That condition could probably be related to SARS-CoV-2, which mainly affects pancreatic islets, harms insulin secretion, and causes further deterioration of hyperglycemia and acute diabetes (17). Also, more than 70% of the reported cases received corticosteroids, which suppress the immune system by hampering the chemotaxis and phagocytosis by leucocytes and reducing the accumulation of inflammatory cells and pro-inflammatory stimuli (16). The steroids elevate blood sugar levels and cause glycosylation of transferrin and ferritin, which decreases binding to iron and allows increased free iron in circulation. In its wake, steroids inhibit the transcription of cytokine genes, which are the stimulating factors for ferritin. 

Therefore, all of the above-mentioned factors interplay in vascular endothelial injury onset and progression and finally could lead to the pathogenesis of mucormycosis (4,5). 

Other important predisposing factors included hypertension (33%) and organ transplant (11%). Nagalli* et al.* reported a suspected link between hypertension and CAM infection (29). As this subject is fairly new, more studies need to be done to determine the stringent role of hypertension as a potential risk factor for CAM infection. A trinity of diabetes, indiscriminate use of steroids, and the background of SARS-CoV-2 infection increased the risk of nosocomial infections including mucormycosis. Previously mucormycosis was regarded as a community-acquired disease but after the COVID-19 pandemic, there have been rising reports of hospital-associated mucormycosis (1,4). Contaminated bandages, mechanical ventilation, catheters or intravascular devices, and adhesive dressings have been implicated in developing cutaneous mucormycosis and have been reported as the origin of the quick spreading of the disease among hospitalized patients (15). In the analysis of 693 cases of mucormycosis from the systematic review by Skiada* et al.*, 108 (15.6%) cases were healthcare-associated mainly due to adhesive tapes, bandages, and intravenous or arterial catheters (28). In a study by Saad* et al.*, there was a report of SCM infection due to the rhino-orbital mucormycosis extension at the site of the non-invasive ventilation (NIV) mask, causing damage and progressive necrosis of the tissues to the left cheek. In this case, the patient was a young man with well well-immune condition with no history of trauma or primary diseases (20). 

Based on the clinical documents, 14 (52%) and 13 (48%) PCM and SCM cases were reported, respectively. Our review indicated that the most frequent anatomic locations of the ulcer among PCM cases were the upper extremities (64.2%) followed by the lower extremities (21.4%) and face (14.2%). Additionally, clinical lesions in all SCM cases occur subsequently to rhino-orbital form extending into the adjacent structures that manifest with eye symptoms, sinus involvement, nasal stuffiness, and necrosis of the skin over the face. 

Our literature search showed that pathogens in 70.3% of cases were diagnosed by microbiological (KOH & Culture), histopathological (55.5%), and molecular (26%) methods. Mucor and Rhizopus are responsible for the reported genera, although in one patient the causative agent was identified as Lichtheimia ramose (14). The R. oryzae is the most common isolate responsible for human mucormycosis that may demonstrate in vitro resistance to posaconazole. 

It is strongly recommended to specifically identify the species of the causative microorganism, due to the different properties between the species with antifungal susceptibility testing (30). 

The usual antifungal treatment choice is amphotericin B as the first line, but it is not effective in many cases, especially if the disease is detected at the late stages or disseminated (31). The lipid-based amphotericin B, despite its high cost, is a better alternative to conventional formulations with potentially severe side effects. Generally, azoles show a tendency to have an increased rate of recurrence and have variable activity against Mucorales. Some Mucor species evidence high MIC values to posaconazole, isavuconazole, and itraconazole than those for Rhizopus and the Lichtheimia (30,32). Also, posaconazole is an alternative therapy option but some Mucorales species demonstrate high MIC values to posaconazole, for example, Lichtheimia corymbifera exhibits resistance to this antifungal (MIC ≥16 μg/mL) (6). 

In a review study, most of the patients had no report of antifungal susceptibility testing and the cases were sensitive to amphotericin B, itraconazole, voriconazole, and posaconazole. Three patients were treated with conventional AmB, and the majority (73%) of them received lipid-based AmB. In several patients, other antifungals alone or in combination were considered effective for the treatment and one case died before treatment (24). Debridement could be necessary to prevent the progression through non-necrotic areas and to reduce the fungal loads, which allow for better dissemination of antifungal drugs to deeper organs and prevent the recurrence (15). Among the patients, medical management included antifungal therapy alone (4, 15%), and combination therapy of antifungal and surgery (23, 85%). None of them had surgery treatment alone. The mortality rate among PCM patients was 36% (5 out of 14). Among this group, 4/6 of the patients who received at least two different antifungal agents survived. Only 12 cases had data on the outcomes in SCM patients, and death was reported in 7 (58.3%) patients. A total of 12 patients died, 75% mortality was reported in diabetic patients, and 92% mortality was reported among steroid users. The patients who received steroids had significantly higher mortality than survival rates. Although, most cases were males, the incidence of mortality was not remarkably different between male (8) and female patients (4), and the mortality mean age in the patients was 53 ± 11.7 years. In PCM, trauma or rupture of the skin barrier is a known trigger. In contrast, it was the most common predisposing factor in our study (5 out of 12, 36%), while 3 patients after recovering from COVID-19 without any preceding trauma, injury, evidence of secondary dissemination, and known modes of transmission at the affected site were diagnosed as primary cutaneous mucormycosis. In four cases and probably including our case, the etiology of primary cutaneous mucormycosis was healthcare-associated.

## Conclusion

Early diagnosis of mucormycosis and prompt intervention is essential to limit the cutaneous mucormycosis manifestations during outbreaks like the COVID-19 pandemic. Moreover, medical treatment with a combination of at least two different antifungal agents may be significantly better than monotherapy. Multiple factors including hyperglycemia, use of long-term antibiotics and corticosteroids, viral-induced lymphopenia, and contaminated equipment play a significant role in the cutaneous mucormycosis development in COVID patients. The use of surgical debridement in combination with antifungal therapy and correction of the underlying debilitating conditions can improve clinical outcomes and limit the mortality rate. Accordingly, cutaneous mucormycosis escalates the need for frequent follow-up in all patients, even among healthy individuals with suspicious signs and symptoms.
